# The Role of IL-22 in Wound Healing. Potential Implications in Clinical Practice

**DOI:** 10.3390/ijms23073693

**Published:** 2022-03-28

**Authors:** Roxana Delia Zaharie, Călin Popa, Diana Schlanger, Dan Vălean, Florin Zaharie

**Affiliations:** 1 Gastroenterology Department, “Iuliu Haţieganu” University of Medicine and Pharmacy, Street Emil Isac No. 13, 400023 Cluj-Napoca, Romania; roxanadeliazaharie@gmail.com; 2 Gastroenterology Department, Regional Institute of Gastroenterology and Hepatology “Prof. Dr. O. Fodor”, Street Croitorilor No. 19–21, 400162 Cluj-Napoca, Romania; 3 Surgery Department, “Iuliu Haţieganu” University of Medicine and Pharmacy, Street Emil Isac No. 13, 400023 Cluj-Napoca, Romania; valean.d92@gmail.com (D.V.); florinzaharie@yahoo.com (F.Z.); 4 Surgery Department, Regional Institute of Gastroenterology and Hepatology “Prof. Dr. O. Fodor”, Street Croitorilor No. 19–21, 400162 Cluj-Napoca, Romania

**Keywords:** interleukin-22, wound healing, tissue repair

## Abstract

Wound healing is a complex process that is mediated and influenced by several cytokines, chemokines, and growth factors. Interleukin-22 (IL-22) is a cytokine that plays a critical role in tissue regeneration. Our study is a systematic review that addressed the implications of IL-22 in the healing of wounds caused by external factors. Thirteen studies were included in our review, most of them being experimental studies. Three clinical studies underlined the potential role of IL-22 in day-to-day clinical practice. IL-22 plays a central role in wound healing, stimulating the proliferation, migration, and differentiation of the cells involved in tissue repair. However, overexpression of IL-22 can cause negative effects, such as keloid scars or peritoneal adhesions. The results of the presented studies are promising, but further research that validates the roles of IL-22 in clinical practice and analyzes its potential implication in surgical healing is welcomed.

## 1. Introduction

An effective tissue repair is essential for proper healing of acute and chronic wounds, as well as for a proper recovery after surgical interventions. A wound is defined as a disruption of the normal anatomical structure and function of a certain tissue; wounds can arise either from internal processes (inflammatory diseases) or external factors (surgical procedures or accidents). The wound healing process consists of four different phases, that succeed chronologically: (1) coagulation and hemostasis, (2) inflammation, (3) proliferation, and (4) wound remodeling, with every phase playing an essential role [1,2]. While tissue regeneration is possible in some species, in humans, wound healing generates a fibroproliferative response, including cell proliferation, migration, differentiation, and remodeling, with scar tissue formation. Pathological wound healing can be expressed by either overproduction of scar tissue, resulting in keloid scars and adhesions, or failure to heal, resulting in persistent chronic wounds and surgical complications, such as anastomotic dehiscence or anastomotic leaks [3]. 

Wound healing is a complex process that involves the local cell environment, immune inflammatory cells, and diverse signaling pathways. The repair process is mediated and co-ordinated by various growth factors, cytokines, and chemokines; these factors are involved in each step of wound healing, and their regulation and dynamic assure the formation of a normal scar [4,5,6]. Identifying factors with specific roles in tissue repair and understanding their dynamic can be an important step toward a better control of the wound healing process; this might be of great use, especially in high-risk patients, such as immunosuppressed or diabetic patients. Interleukin-22 (IL-22) is a member of the interleukin-10 cytokine family, being produced by several immune cell types [7,8]. IL-22 has been identified as an important regulator in tissue regeneration and wound healing [8], with the extent of its effects still being investigated. 

We intended to perform a systematic review on the role of IL-22 in wound healing, with an emphasis on external wound healing. IL-22 seems to have essential roles in tissue repair, but not a lot of data are available, while the existent information is quite heterogenous and inconsistent. Therefore, we believe that an overview of the medical literature on the subject would be useful for clinicians, and for researchers as well. We believe that a good systematization of the effects of IL-22 on wound healing will highlight the main ideas that should be further investigated and that can have potential applications in clinical practice. 

## 2. Materials and Methods

A systematic review was designed, conducted, and reported based on the Preferred Reporting Items for a Systematic Review and Meta-analysis of Individual Participant Data criteria (PRISMA) [9,10]. The systematic review was conducted based on a previously designed protocol, the details of which will be presented below.

This systematic review intended to reunite the existing data published in medical literature regarding the implications of interleukin-22 in the process of wound healing, referring to wounds generated by external factors, such as trauma, accidents, or surgery. 

We included original research studies, either experimental or clinical studies, with the focus on the role of IL-22 in the external wound healing process. We included retrospective or prospective design studies, cohort studies, comparative studies, trials, case reports, or case series. We excluded commentary articles, reviews, editorials, and articles written in languages other than English. We intended to focus on original articles, which analyzed the specific roles of IL-22 and provide proved and objective results. We believe that commentary articles and editorials might present a subjective opinion and do not provide detailed methodology and results, so a proper interpretation of the data cannot be carried out; therefore, we decided not to include these types of papers in our review. Moreover, we did not include other review articles, since they provide a synthesis and interpretation of the primary data; however, we did search the reference list of these reviews in order to see if we can identify other articles that respect our inclusion and exclusion criteria. We also excluded articles with other topics or a different focus, such as those referring to wounds caused by internal disruptions, for example, inflammatory bowel diseases or inflammatory or degenerative skin diseases. 

We conducted a systematic search of three databases: Pubmed, Embase, and Scopus, as well as the references lists of the included articles and the other identified review articles. The following terms were used for the search: “interleukin-22” or “IL-22” and “wound repair” or “wound healing” or “tissue repair”. No automation tools were used for the search, with all identified papers being analyzed individually. The date of the last search was January 2022. No time restrictions were imposed for the search. In the first stage, the results were first screened based on their title and abstract, verifying that they respect the inclusion and exclusion criteria, by two different reviewers (D.S., C.P.). In the second stage, we further analyzed the selected articles based on their full text and excluded the ones that do not respect the above-mentioned criteria. The articles selected after our two-stage process were included in our systematic review. Any discrepancies were resolved by consensus or by consulting a third reviewer (DV). 

The data were collected from the included articles, using spreadsheets that gathered much information from the included studies: title and author name, year of publication, type of study, study design, study population, main outcomes and endpoints, limitations, potential clinical implications, and conclusions. Afterwards, the extracted data were synthetized in tables. 

A high variability was observed between studies, from their scope and objective to the study design and data interpretation. Therefore, a meta-analysis was ruled out, the results being described narratively. 

## 3. Results

### 3.1. Search Strategy

**Figure 1:**
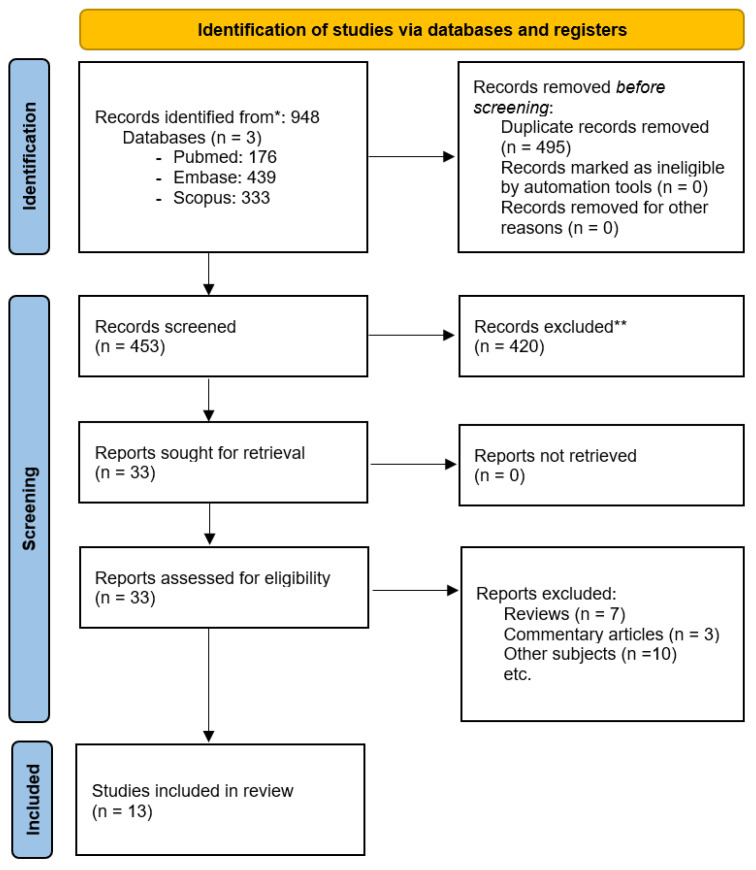
Search strategy—PRISMA flow diagram. * Consider, if feasible to do so, reporting the number of records identified from each database or register searched (rather than the total number across all databases/registers). ** If automation tools were used, indicate how many records were excluded by a human and how many were excluded by automation tools.

We identified a total of 948 articles (176 articles in the Pubmed database, 439 articles in the Embase database, and 333 articles in the Scopus database). We have identified and excluded 495 duplicate records. The remaining records were screened based on their title and abstract, 420 being excluded. No automation tools were used in the exclusion process, all articles being assessed by the investigators. Finally, 33 records were assessed based on the full-text article and 13 articles that fulfilled the proposed criteria were included in our review. The search strategy is described in Figure 1. 

### 3.2. Included Studies 

The included studies are listed in Table 1. All studies were published in the last decade, from 2011 to 2021, with most of the studies being published in the last 5 years (nine studies—69.23%). The articles analyzed the healing of different types of wounds, from intestinal damage to skin wounds or corneal lesions. Most studies (eight studies—61.53%) are experimental animal studies, two studies are clinical human studies (15.38%), one study has a mixed design, consisting in an experimental part and a clinical part (7.69%), and, finally, two studies were based on cell cultures (15.38%). 

#### 3.2.1. Cell Culture Studies 

Two studies were based on cell cultures, one of them used colon cells [14] and the other used keratinocytes [21]. They investigated the role of one cell population on the repair of intestinal and skin wounds, respectively. Both studies reported a positive role of IL-22 in tissue repair by inducing the proliferation or migration of the specific investigated cells. (Table 2)

#### 3.2.2. Experimental Studies 

The experimental studies are described in Table 3, along with the type of wound studied, the experimental animal, the endpoint of the study, and the implication of IL-22. We included in this subcategory the one study with mixed clinical and experimental design [15], referring only toward the experimental part of the study; the clinical study will be discussed in the respective subcategory. Three studies [18,20,22] referred to skin wounds (33.33% out of nine experimental studies), one [19] to oral mucosa injury (11.11%), and another [23] to corneal abrasion (11.11%), while one [13] study discussed intestinal lesions (11.11%), one [12] study analyzed colon and skin lesions (11.11%), one [15] study analyzed peritoneal adhesions formation (11.11%), and another study [11] referred to appendix (limb/tail) amputations (11.11%). One study [11] used lizards as experimental animals (for their differential wound healing in two different body parts—limb versus tail), while the others utilized mice. All studies except one [13] reported the positive involvement of IL-22 in tissue repair; one study [13] reported IL-22-independent healing pathways in intestinal lesions. 

#### 3.2.3. Clinical Studies

Three studies have been conducted in clinical settings (Table 4); one study [15] with a mixed study design was also reported in the experimental study paragraph. The samples used for analysis varied from skin biopsy to drainage fluid (peritoneal or cervical), all being local samples and not systemic. All studies were observational studies, two [16,17] of them having a comparative design. One study [16] did not find a significant role for IL-22 in wound healing, other cytokines being attributed a significant implication; this study referred to healing after lymph node resection for cervical tuberculosis. 

## 4. Discussion

### 4.1. Current Context and Importance 

The role of the IL-22 in tissue repair has been recently reported, particularly in mucosal healing in inflammatory bowel disease. Its role in wounds generated by external factors, such as accidents, trauma, or surgery, has been less analyzed. Proper tissue healing in such cases is extremely important, especially after surgical interventions; delayed wound healing or failure to heal is responsible for severe complications. Anastomotic dehiscence or leaks are one of the most feared complications after digestive surgery, having a massive impact on postoperative mortality, functional outcomes, quality of life, and hospital resources. While there are some known modifiable factors that can lower the risk of such complications (for example, nutritional status), there is still a lack of consensus regarding the exact factors that might predispose to anastomotic leakage and insufficient knowledge towards the molecular factors that influence the process. The complexity of the wound healing process makes it vulnerable at many levels, being influenced by local factors (infection, ischemia, etc.) or by systemic factors (age, diabetes, hypothyroidism, etc.) [8]. 

While the studies discussing the implication of IL-22 in tissue repair in the case of inflammatory bowel diseases [7,24,25] offer insight regarding how IL-22 stimulates the regeneration of the injured bowel, the results are not completely applicable to other types of wounds, such as surgical wounds, for example, due to the completely different pathophysiology between these two cases. 

This systematic review intended to perform a thorough search of the medical literature to identify and to synthetize the main ideas on the role of IL-22 in external wound healing, keeping in mind the potential applicability in clinical practice.

An objective of our study was to be helpful for other researchers exploring the topic of wound healing and the specific role of IL-22 in this process, since it provides an overview of the current publications and the topics which need more exploration. For clinical researchers, more specifically, our paper highlights the most appealing implications of IL-22 that should be further studied. 

### 4.2. The Wound Healing Process 

A better understanding of the molecular environment of the wound healing process allowed the identification of key factors involved, interleukin-22 being one of these factors. For a clear perspective regarding our paper, a proper overview of the physiology of the normal and pathological wound healing process is necessary.

- Coagulation and inflammation phase

Vasoconstriction is triggered in the wounded area and a fibrin blood clot plug is formed by the initiation of the clotting cascade. The fibrin plug will play the role of a matrix. Blood platelets play an important role, releasing growth factors and cytokines (mainly platelet-derived growth factor and transforming growth factors), which attract inflammatory cells: neutrophils and macrophages. These cells have a role in antimicrobial defense. Resolution of the inflammatory phase is determined by the apoptosis of inflammatory cells, which occurs gradually, guided by anti-inflammatory cytokines. Therefore, the resolution of the inflammatory phase induces the next phase by releasing growth factors and cytokines that stimulate proliferation: interleukin 1, interleukin 6, tumor necrosis factor-α, platelet-derived growth factor, and fibroblast growth factor-2 [26,27,28,29,30,31].

- Proliferation phase

This phase is characterized by granulation tissue formation and angiogenesis for restoring the vascular supply. Various molecular factors stimulate cell proliferation and the angiogenic response: the transforming growth factor family, the interleukin family, vascular endothelial growth factor, platelet-derived growth factor, and fibroblast growth factor [26,27,28,29,30,31].

- Remodeling phase

Remodeling is the final phase of the wound healing process, which assures the maturation of the previously formed granulation tissue, ending up with scar formation. In this phase, the angiogenic response ceases, the extracellular matrix is remodeled, and wound contraction begins [26,27,28,29,30,31]. 

As presented above, wound healing is a complex process composed of a cascade of biological events that guide cellular responses. Thorough research has been able to describe and identify the specific role of many molecules involved, but a lot of pathways and key factors remain unelucidated. IL-22 is a recently described IL-10 family cytokine, widely spread in different types of tissues, that seems to promote cell proliferation and, therefore, being involved in tissue regeneration [32]. Not enough is known regarding the role of IL-22 in wound healing, as shown by our systematic review focusing on this fresh topic, which might bring new information regarding a key factor in this process.

Any disruption in the wound healing phases leads to excessive wound healing (hypertrophic or keloid scars) or chronic wound formation (failure to heal). Numerous factors, such as age, immune status, malnutrition, infection, ischemia, smoking, diseases, and medication, affect the wound healing process [29,33,34,35]. Impaired wound healing in the aged is associated with altered inflammatory response, but also delayed epithelization, collagen production, and remodeling [29,36,37,38]. Regarding the diseases that affect the wound healing process, diabetes is one of the most common pathologies involved, impaired healing being caused by hypoxia, dysfunction of fibroblasts and epidermal cells, impaired angiogenesis, and decreased host immune response [29,33,34,39].

Highlighting the role of IL-22 in the healing of external generated wounds does not only provide a better understanding of this biological process, but, most importantly, identifies the potential clinical applications of this role. Therefore, IL-22 might be used for identifying high-risk patients for delayed wound healing or it might be manipulated in order to improve tissue repair. 

### 4.3. Overview of Literature

The literature is scarce on our proposed topic, but the initial results are promising. As we mentioned before, most studies are experimental in design, and are either animal studies or studies on cell cultures, which is somewhat expected for such a novel topic. 

The studies involving cell cultures reported that IL-22 has effects on intestinal epithelial cells, promoting their proliferation [14], and on keratinocytes, promoting their migration [21]. One study, by Moniruzzaman et al. [14], evaluated the potential of three cytokines from the IL-20 family to promote colonic epithelial cell proliferation. A live-cell-imaging system showed that only IL-22, and not IL-20 and IL-24, significantly promoted the proliferation of intestinal cells. This study also reported that IL-22 increases oxidative stress and reduces the secretory function of the affected cells. This indicates that IL-22 does not only stimulate cell proliferation, but also affects the cell’s behavior. Huang et al. [21] used keratinocytes treated with conditioned media released from mononuclear cells, cultivated in various glucose environments. Keratinocyte migration was impaired when using high-glucose mediums; it was observed that IL-22 expression was reduced in these conditions. The essential role of IL-22 was also demonstrated by observing a reduction in keratinocyte migration in normal glucose environments when IL-22 binding protein was used. Therefore, this study highlights that the impaired wound healing observed in diabetic patients might be determined by the reduced production of IL-22. IL-22 can induce tissue repair by stimulating the local cells. While these studies highlight the specific effect that IL-22 has on a certain type of cell, they do not properly assess the effect on the wound microenvironment, therefore simplifying the process. The described effects of IL-22 should be replicated in animal and human studies in order to confirm their validity. 

The experimental animal studies focus on identifying the different roles of IL-22 in tissue repair, describing also the underlying pathophysiological pathways and mechanisms. Although skin wounds [12,18,20,22] were the most common topic (the focus in 44.44% of the nine experimental design studies), reports on intestinal [12,13], oral mucosal [19], and corneal [23] wounds are also available, showing the extent of IL-22 effects on different types of tissues. All studies agree that IL-22 plays a central role in the healing process, although one study [13] reports an IL-22-independent way through which intestinal stem cell proliferation is induced. In skin wound repair, IL-22 exerts its positive roles by stimulating both the keratinocytes [20] and the fibroblasts [22]. Besides these effects, one study also showed improved wound contraction and different composition of the granulation tissue (higher cellularity and higher collagen proportion) in IL-22-treated wounds [22]. The involvement of IL-22 in skin wound repair is seen early in the process, due to the inflammatory phase: the inflammatory response to injury is responsible for inducing the expression of IL-22 and of initiating the tissue regeneration by stimulating cell proliferation [12]. Oral mucosa repair is promoted by stimulating the migration and proliferation of the keratinocytes [19]. In corneal lesions, IL-22 stimulates the division of epithelial cells, but also plays an important role in guiding the inflammatory response, by assuring the influx of neutrophils and platelets in the wound area [23]. Regarding intestinal injury, both IL-22-dependent and IL-22-independent pathways seem to have important roles in tissue repair [12,13]. One interesting report [11] shows that IL-22 plays a role in both restorative scarless healing and healing through scar tissue in lizards, the cytokine constellation guiding each type of healing. In summary, the experimental studies show that IL-22 mediates the interaction between immune cells and local cells involved in the proliferation and remodeling phase of wound healing. IL-22 has effects that extend beyond influencing the epithelial cells (especially keratinocytes in skin wounds), since it also stimulates fibroblasts and directs extracellular matrix regeneration, having actions on multiple levels of the healing process. 

The three studies with a clinical component are valuable and offer a small preview of the possibilities of day-to-day clinical practice applications. The clinical studies only refer to skin healing and peritoneal adhesion formation. Wang et al. [15] is the only study that focused on the topic of postoperative peritoneal adhesions; the investigators used an experimental animal model, as well as a clinical model, both showing the rapid rise in IL-22 levels in the drainage fluid in the first 24 h after surgery, with a slow decrease afterwards. The normal state of the abdominal cavity showed no expression of IL-22, since, at incision, the cytokine’s levels were almost null. In the experimental model, the investigators attempted to block the action of IL-22 in the mouse model, obtaining a significant reduction in adhesion formation when the neutralization by intraperitoneal injection of anti-IL-22 antibodies was performed 3 to 5 days after surgery. Therefore, blocking the expression of IL-22 in a certain timeframe after surgery can potentially reduce the formation of postoperative adhesions and, consequently, the complications induced by them. The clinical studies investigating skin healing analyzed the wound healing process in patients with cervical node tuberculosis undergoing surgical resection [16] and keloid scar formation [17]. Regarding the healing after cervical lymphadenectomy [16], there was no significant difference between the IL-22 levels in the drainage fluids between normal and delayed healing scars (only IL-6 and VEGF-A levels differed); however, compared to the control group of patients undergoing thyroidectomy, patients with cervical lymph node tuberculosis had higher concentrations of IL-22. These results show that the cytokine expression is disease-dependent. However, in cervical node tuberculosis, IL-22 does not play the central role in wound healing. Another article [17] reported a significantly higher local expression of IL-22 mRNA in keloid scars. Biopsies from several normal and keloid scars were collected and the mRNA expression of several growth factors and cytokines were determined: transforming growth factor-β, fibroblast growth factor, IL-33, IL-22, arginase-1, arginase-2, inducible nitric oxide synthase, vasoactive intestinal peptide, and its receptor. Only IL-22, transforming growth factor-β, and arginase-1 exhibited significant higher levels in keloid scars. The overexpression of IL-22, together with other molecule alterations, contribute to pathological scarring. This study also underlines the fact that the dynamic of multiple molecular factors guides the normal wound healing process, and that the interactions between them should also be taken into consideration when interpreting the conclusions. The studies of da Cunha et al. [17] and Wang et al. [15] underline the fact that, although IL-22 acts like a promoter of wound healing, the overexpression of IL-22 can cause defective healing, such as keloid scars or peritoneal adhesions. 

### 4.4. Main Roles of IL-22 in Wound Healing

Based on the articles included in our systematic review, we extracted the main roles that IL-22 plays in wound repair, which will be resumed next, in order to provide a short and structured summary:

- IL-22 accelerates wound healing in the skin, digestive mucosa (oral, intestinal), and cornea:
  ○ Induces epithelial cells migration and division;
  ○ Increases proliferation, migration, and differentiation of keratinocytes;
  ○ Induces myofibroblast differentiation;
  ○ Stimulates extracellular matrix production;
  ○ Contributes to adequate wound contraction and tissue remodeling.
- Overexpression of IL-22 determines formation of pathological scars, such as keloid scars.
- Increased levels of IL-22 are responsible for peritoneal adhesion formation.

### 4.5. The Restorative Potential of IL-22 on Different Tissues

#### 4.5.1. Skin 

IL-22 has positive roles, such as stimulating tissue repair, even in cases of challenging wounds (for example, in diabetic patients [21] or patients diagnosed with lymph node tuberculosis [16]). On the other hand, pathologic scarring is also a consequence of IL-22 expression, with high levels of these cytokine being identified in keloid scars [17]. IL-22 expression is induced in the inflammatory phase [12,22], after acute skin injury, and stimulates the proliferation and migration of not only epithelial cells [21], but also influences the skin’s dermal compartment by stimulating fibroblasts and extracellular matrix formation [22]. 

Skin wounds are frequently encountered and external factors are mostly incriminated for their occurrence. In surgery, skin wounds are encountered from minor, superficial interventions to major abdominal surgery, skin wound repair being an important component of the patient’s recovery, even though it was not the focus of the intervention. Therefore, it is not surprising that skin wounds are the most discussed topic in the identified studies. 

#### 4.5.2. Mucosa

Like in skin injuries, IL-22 has a positive involvement in mucosal wound healing, inducing local cell proliferation. In intestinal injury repair, there are also important mechanisms that do not depend on IL-22. The evidence on intestinal wound healing is scarce, most of the ideas being indirect, deducted conclusions. Moreover, the role of IL-22 in intestinal wound repair can be deducted from its important role in regeneration in inflammatory bowel diseases [7,24].

No study directly discussed the implications of IL-22 in healing after a gastrointestinal anastomosis; this subject is of great importance and can be explored in experimental and clinical settings as well. As mentioned before, anastomotic leaks deeply affect the postoperative evolution of patients. Anastomoses are widely used in digestive surgery, a variety of surgical techniques being available (from mechanical anastomosis to hand-sewn anastomosis), but leaks are encountered in every site and associated with different techniques used. Since anastomotic leaks severely influence surgical morbidity and IL-22 has been proved to stimulate epithelial cell proliferation and intestinal tissue repair, we believe that this subject deserves more interest. 

#### 4.5.3. Cornea

The evidence for the role of IL-22 in corneal healing is little, with only one published study [23] discussing this topic. The results suggest that IL-22 is implicated in the inflammatory response after corneal abrasion and promotes epithelial healing.

The cornea, formed by a stratified epithelium, has an important role in visual clarity; the proper healing of this tissue is important in order to maintain its role. Although only one study has discussed the subject, IL-22 seems to be a promoter of efficient corneal healing. 

#### 4.5.4. Peritoneum

After peritoneal surgical injury, IL-22 is overexpressed and seems to be related to adhesion formation [15]. This hypothesis is supported by the fact that blocking IL-22 expression results in fewer postoperative adhesions.

Postoperative peritoneal adhesions are a common complication that can occur after any abdominal surgery. The exact mechanism of this pathology and the factors involved are not clearly understood yet. Extensive adhesion formation can be responsible for other surgical complications, such as intestinal obstruction, which might necessitate reintervention. The importance of this subject should not be underestimated but, unfortunately, only one [15] study explored this subject. Learning the fact that the formation of peritoneal adhesions might be manipulated by adjusting the expression of IL-22 can be very useful in clinical practice. 

### 4.6. Study Limitations

All included studies bring novel information, but some limitations do exist, and it is important to underline them, for creating a fair assessment of the current topic. Most studies have experimental design, so their results need to be validated and replicated in clinical studies in order to assess their reliability in day-to-day clinical practice. Some studies have a few flaws in their methodology, making the validity of their results questionable. For example, one study [17] analyzed the expression of cytokines in keloid scars after preoperative corticosteroid treatment, which may have altered the inflammatory response and cytokine expression. Another study [14], which used cell cultures, utilized human adenocarcinoma cells for analysis; malignant cells may have different dynamics than normal cells; this fact needs to be taken into consideration when interpreting the results. Moreover, when considering the role of IL-22 in wound repair, the interaction with other cytokines needs to be taken into discussion; not all studies [14,15,19,20,21,22] considered these possible interactions. 

Having these limitations in mind, we still need to acknowledge the importance of the included studies, which bring exciting information and, thus, encourage the further exploration of this subject. 

### 4.7. Future Research Opportunities

We believe that the presented studies show unequivocally that IL-22 has an important role in wound healing. Therefore, we consider that the most important direction to follow is to validate the results in clinical practice. Nonetheless, the further exploration of IL-22 on surgically inflicted wounds is mandatory, since it may play an essential role in anastomotic healing or development of leaks, anastomotic stenosis, adhesion formation, surgical wound healing, and so on. More specifically, the healing of gastrointestinal anastomosis should be a new focus in investigating the role of IL-22, while peritoneal adhesion formations should be explored more. Surgical applications might be the most underrated topic when discussing IL-22 dynamics; we believe that this is a valuable topic that invites more exploration.

We intended, through our work, to be of help to researchers that wish to investigate the proposed subject, offering an in-depth perspective of the current available information, while highlighting the needs of future research. 

### 4.8. Potential Roles in Clinical Practice

Before discussing the applicability of IL-22 research on wound healing in clinical practice, more research needs to be carried out, especially clinical studies performed on human subjects. 

Based on the information we have available today, we can already speculate some future possibilities regarding the clinical applications of IL-22:

- Identifying high risk patients for poor wound healing;
- Identifying patients at risk for developing anastomotic leaks;
- Prevention of peritoneal adhesions;
- Effective treatment of chronic wounds;
- Promoting wound healing in diabetic patients.

## 5. Conclusions

IL-22 plays an important role in guiding wound healing in different types of tissues. Experimental studies show promising results, highlighting the beneficial roles of IL-22 in wound repair, and the few clinical studies sustain these results. More clinical studies are necessary for validation of these effects and guiding its possible uses in clinical practice. 

## Figures and Tables

**Table 1:** Studies included in the systematic review.

No.	Study	Author	Year	Study Type
1	Site-specific variation in the activity of COX-2 alters the pattern of wound healing in the tail and limb of northern house gecko by differentially regulating the expression of local inflammatory mediators [11]	Khaire et al.	2021	Experimental animal study
2	GPR34-mediated sensing of lysophosphatidylserine released by apoptotic neutrophils activates type 3 innate lymphoid cells to mediate tissue repair [12]	Wang et al.	2021	Experimental animal study
3	Yap1-Driven Intestinal Repair Is Controlled by Group 3 Innate Lymphoid Cells [13]	Romera-Hernandez et al.	2020	Experimental animal study
4	Interleukin (IL)-22 from IL-20 Subfamily of Cytokines Induces Colonic Epithelial Cell Proliferation Predominantly through ERK1/2 Pathway [14]	Moniruzzaman	2019	Cell culture study
5	Regulation and function of IL-22 in peritoneal adhesion formation after abdominal surgery [15]	Wang et al.	2019	Experimental animal study + Clinical study
6	High IL-6 and VEGF-A levels correlate with delayed wound healing in cervical lymph node tuberculosis patients [16]	Wang et al.	2018	Clinical study
7	High in situ mRNA levels of IL-22, TFG-β, and ARG-1 in keloid scars [17]	da Cunha Colombo Tiveron et al.	2018	Clinical study
8	IL-22R Ligands IL-20, IL-22, and IL-24 Promote Wound Healing in Diabetic db/db Mice [18]	Kolumam et al.	2017	Experimental animal study
9	IL-22 mediates the oral mucosal wound healing via STAT3 in keratinocytes [19]	Yu et al.	2016	Experimental animal study
10	Interleukin-22 Promotes Wound Repair in Diabetes By Improving Keratinocyte Pro-Healing Functions [20]	Avitabile et al.	2015	Experimental animal study
11	High-glucose-cultivated peripheral blood mononuclear cells impaired keratinocyte function via reduced IL-22 expression: implications on impaired diabetic wound healing [21]	Huang et al.	2015	Cell culture study
12	Interleukin-22 promotes fibroblast- mediated wound repair in the skin [22]	McGee et al.	2012	Experimental animal study
13	CCL20, γδ T cells, and IL-22 in corneal epithelial healing [23]	Li et al.	2011	Experimental animal study

**Table 2:** Cell culture studies included in the systematic review.

No.	Cell Type	Study Endpoint	IL-22 Role
4 [14]	Cultured human colon adenocarcinoma cells	The potential role of IL-20, IL-22, and IL-24 in colonic epithelial renewal	IL-22 induces cell proliferation in highly proliferative cells, such as intestinal epithelial cells.
11 [21]	Cultured keratinocytes	The function of mononuclear cells in the re-epithelialization process of diabetic wounds	High-glucose cultivation reduced the IL-22 production in mononuclear cells, hampering the migration of keratinocytes.

**Table 3:** Experimental studies included in the systematic review.

No.	Wound Site	Experimental Animal	Study Endpoint	IL-22 Role
1 [11]	Amputation of the tail/limb	Lizard	Differential wound healing—regenerative healing in tail versus scarring in limbs	IL-22 is involved in both processes, in synergy with IL-17
2 [12]	Skin Colon	Mice	The role of apoptotic neutrophils in tissue repair	IL-22 has a role in epithelial repair in both intestinal and skin injuries
3 [13]	Intestinal	Mice	The role of Group 3 Innate Lymphoid Cells in tissue repair	Crypt Cell Proliferation after Stem Cell Damage is IL-22 Independent
5 [15]	Peritoneum	Mice	The function of IL-22 in peritoneal adhesions formation	IL-22 promoted adhesion formation
8 [18]	Skin	Mice	The effects of IL-20 family citokines on skin healing	IL-22 fusion protein was able to accelerate wound healing
9 [19]	Oral mucosa	Mice	The function of IL-22 in oral mucosal wound healing	IL-22 induces proliferation and migration of keratinocytes and contributes to scarless healing
10 [20]	Skin	Mice	The response of diabetic keratinocytes to IL-22 treatment	IL-22 modulates keratinocyte differentiation and VEGF production
12 [22]	Skin	Mice	The role of IL-22 in skin wounds healing	IL-22 induces myofibroblast differentiation and extracellular matrix production
13 [23]	Cornea	Mice	The role of IL-22 in corneal healing	IL-22 promotes corneal epithelial healing

**Table 4:** Clinical studies included in the systematic review.

No.	Study Type	Analyzed Samples	Study Endpoint	IL-22 Role
5 [15]	Cohort observational study	Peritoneal drainage fluid	The function of IL-22 in peritoneal adhesions formation	IL-22 promoted adhesion formation
6 [16]	Comparative observational study	Cervical drainage fluid	Cytokines and growth factors levels in slow versus fast wound healing	High IL-6 and VEGF-A levels in the postoperative wound fluid of cervical lymph node tuberculosis patients correlate with delayed wound healing.
7 [17]	Comparative observational study	Skin biopsy	In situ mRNA expression of growth factors and cytokines in keloid and normal scars	Significant high expression of TGF-ß, IL-22, and ARG-1 was found in keloid scars

## Data Availability

Not applicable.
